# Some generalizations of the new SOR-like method for solving symmetric saddle-point problems

**DOI:** 10.1186/s13660-018-1738-3

**Published:** 2018-06-26

**Authors:** Ruiping Wen, Ruihuan Wu, Jinrui Guan

**Affiliations:** 1 Key Laboratory of Engineering & Computing Science, Shanxi Provincial Department of Education/Department of Mathematics, Taiyuan Normal University, Jinzhong, P.R. China; 2 Department of Mathematics, Taiyuan Normal University, Taiyuan Normal University, Jinzhong, P.R. China

**Keywords:** Saddle-point problems, SOR-like method, Matrix splitting iteration method, Convergence

## Abstract

Saddle-point problems arise in many areas of scientific computing and engineering applications. Research on the efficient numerical methods of these problems has become a hot topic in recent years. In this paper, we propose some generalizations of the new SOR-like method based on the original method. Convergence of these methods is discussed under suitable restrictions on iteration parameters. Numerical experiments are given to show that these methods are effective and efficient.

## Introduction

Consider the solution of the following symmetric saddle-point linear system with block 2-by-2 structure:

1.1 $$ \mathcal{A}x\equiv \left ( \textstyle\begin{array}{c@{\quad}c} A & B\\ B^{T} & 0 \end{array}\displaystyle \right ) \left ( \textstyle\begin{array}{c} y \\ z \end{array}\displaystyle \right ) =\left ( \textstyle\begin{array}{c} p \\ q \end{array}\displaystyle \right )\equiv b, $$

where $A\in\mathbb{R}^{m\times m}$ is a symmetric positive definite matrix, $B\in\mathbb{R}^{m\times n}$ is a matrix of full column rank ($m \gg n$), $B^{T}\in\mathbb{R}^{n\times m}$ is the transpose of the matrix *B*, and $p\in\mathbb{R}^{m}$ and $q\in\mathbb{R}^{n}$ are given vectors. Under such conditions, system (1.1) has a unique solution [8]. Problems of this type arise in many areas of scientific computing and engineering applications, such as computational fluid dynamics, constrained and weighted least squares estimation, constrained optimization, image reconstruction, computer graphics, and so on. For background and a comprehensive survey, we refer to [3, 5, 8, 12].

Since the matrices *A* and *B* are usually very large and sparse in applications, the direct methods are not suitable to be applied to solve system (1.1). So, more attention has been paid on the iteration methods for solving problem (1.1). Many iteration methods were found for system (1.1): the Uzawa-type methods [1], matrix splitting methods, Krylov subspace methods, and so on. See [2, 3, 5, 8, 10, 11, 14–17, 19–23, 25–28] for more details. One of the best iteration methods is SOR-like method, introduced by Golub et al. [13], which includes the Uzawa-type methods as special cases. Later, many researchers generalized or modified the SOR-like method and studied their convergence properties for solving problem (1.1) from different points of view in recent years. For instance, Bai et al. [5] proposed a generalized SOR-like method, which has two parameters and is more effective than the SOR-like method; Shao et al. [19] extended this method and proposed a modified SOR-like method, and Guo et al. [15] presented another modified SOR-like method; Zheng et al. [27] also discussed a new SOR-like method based on a different splitting of the coefficient matrix; Darvishi and Hessari [10], Wu et al. [23], and Najafi et al. [18] considered the SSOR method. We refer to [2–28] and the references therein.

Recently, Guan et al. [14] came up with a new SOR-like method (NSOR-like) for solving the following equivalent system (1.2) of problem (1.1):

1.2 $$ \left ( \textstyle\begin{array}{c@{\quad}c} A & B\\ -B^{T} & 0 \end{array}\displaystyle \right ) \left ( \textstyle\begin{array}{c} y \\ z \end{array}\displaystyle \right ) =\left ( \textstyle\begin{array}{c} p \\ -q \end{array}\displaystyle \right ). $$

The coefficient matrix can be split as follows:

$$\mathcal{A}=\left ( \textstyle\begin{array}{c@{\quad}c} A & B\\ -B^{T} & 0 \end{array}\displaystyle \right )=\mathcal{D}- \mathcal{L}-\mathcal{U}, $$

where

1.3 $$ \mathcal{D}= \left ( \textstyle\begin{array}{c@{\quad}c} \frac{1}{\alpha}A & 0 \\ 0 & Q \end{array}\displaystyle \right ), \qquad \mathcal{L}=\left ( \textstyle\begin{array}{c@{\quad}c} 0 & 0 \\ B^{T} & \beta Q \end{array}\displaystyle \right ),\qquad \mathcal{U}=\left ( \textstyle\begin{array}{c@{\quad}c} (\frac{1}{\alpha}-1)A & -B \\ 0 & (1-\beta) Q \end{array}\displaystyle \right ) $$

with a symmetric positive definite matrix $Q\in\mathbb{R}^{n\times n}$ and parameters $\alpha>0$, $\beta\geq0$, and $0<\omega<2$. The following iteration scheme was introduced for solving (1.2):

$$(\mathcal{D}-\omega\mathcal{L})\left ( \textstyle\begin{array}{c} y_{k+1} \\ z_{k+1} \end{array}\displaystyle \right )= \bigl[(1-\omega)\mathcal{D}+\omega\mathcal{U}\bigr]\left ( \textstyle\begin{array}{c} y_{k} \\ z_{k} \end{array}\displaystyle \right )+ \omega \left ( \textstyle\begin{array}{c} p \\ -q \end{array}\displaystyle \right ), $$

or, equivalently,

$$\left ( \textstyle\begin{array}{c} y_{k+1} \\ z_{k+1} \end{array}\displaystyle \right )= \mathcal{M}\left ( \textstyle\begin{array}{c} y_{k} \\ z_{k} \end{array}\displaystyle \right )+\mathcal{N} \left ( \textstyle\begin{array}{c} p \\ -q \end{array}\displaystyle \right ), $$

where

1.4 $$\begin{aligned}[b] \mathcal{M}&= (\mathcal{D}-\omega\mathcal{L})^{-1} \bigl[(1-\omega )\mathcal{D}+\omega\mathcal{U}\bigr] \\ &= \left ( \textstyle\begin{array}{c@{\quad}c} \frac{1}{\alpha}A & 0 \\ -\omega B^{T} & (1-\omega\beta) Q \end{array}\displaystyle \right )^{-1}\left ( \textstyle\begin{array}{c@{\quad}c} (\frac{1}{\alpha}-\omega)A & -\omega B \\ 0 & (1-\omega\beta) Q \end{array}\displaystyle \right ),\end{aligned} $$

and $\mathcal{N}=\omega(\mathcal{D}-\omega\mathcal{L})^{-1}$.

In this paper, we generalize this method to several variants for solving problem (1.1) or (1.2). These methods include some of the above-mentioned methods as particular cases. We discuss the convergence of these methods under suitable restrictions on iteration parameters, which are very easy to use in computations. Numerical experiments are given to show that these methods are effective and efficient.

The rest of the paper is organized as follows. In Sect. 2, we propose several generalizations based on the new SOR-like method for solving problem (1.1) or (1.2). In Sect. 3, we give the convergence analysis of these methods. We use some numerical examples to show the effectiveness of them in Sect. 4. A concluding remark is drawn in Sect. 5.

## Methods

In the section, we derive some generalizations for solving system (1.1) or (1.2) based on the new SOR-like method introduced by Guan et al. [14].

### The new SSOR-like method (NSSOR-like)

By combining the NSOR-like method and its backward version the NSSOR-like method can be easily obtained for solving problem (1.2). The backward iteration scheme of the NSOR-like method is as follows:

$$(\mathcal{D}-\omega\mathcal{U})\left ( \textstyle\begin{array}{c} y_{k+1} \\ z_{k+1} \end{array}\displaystyle \right )= \bigl[(1-\omega)\mathcal{D}+\omega\mathcal{L}\bigr]\left ( \textstyle\begin{array}{c} y_{k} \\ z_{k} \end{array}\displaystyle \right )+ \omega \left ( \textstyle\begin{array}{c} p \\ -q \end{array}\displaystyle \right ), $$

or, equivalently,

$$\left ( \textstyle\begin{array}{c} y_{k+1} \\ z_{k+1} \end{array}\displaystyle \right )= \mathcal{M}_{1} \left ( \textstyle\begin{array}{c} y_{k} \\ z_{k} \end{array}\displaystyle \right )+\mathcal{N}_{1} \left ( \textstyle\begin{array}{c} p \\ -q \end{array}\displaystyle \right ), $$

where

2.1 $$ \begin{aligned}[b]\mathcal{M}_{1}&= (\mathcal{D}-\omega \mathcal{U})^{-1}\bigl[(1-\omega )\mathcal{D}+\omega\mathcal{L}\bigr] \\ &= \left ( \textstyle\begin{array}{c@{\quad}c} \frac{\alpha\omega-\omega+1}{\alpha}A & \omega B \\ 0 & (\omega\beta-\omega+1) Q \end{array}\displaystyle \right )^{-1}\left ( \textstyle\begin{array}{c@{\quad}c} \frac{1-\omega}{\alpha}A & 0 \\ \omega B^{T} & (\omega\beta-\omega+1) Q \end{array}\displaystyle \right ),\end{aligned} $$

and $\mathcal{N}_{1}=\omega(\mathcal{D}-\omega\mathcal{U})^{-1}$. Then the NSSOR-like method can be written as

$$\left ( \textstyle\begin{array}{c} y_{k+1} \\ z_{k+1} \end{array}\displaystyle \right )= \mathcal{T}_{1} \left ( \textstyle\begin{array}{c} y_{k} \\ z_{k} \end{array}\displaystyle \right )+\mathcal{C}_{1} \left ( \textstyle\begin{array}{c} p \\ -q \end{array}\displaystyle \right ), $$

where $\mathcal{T}_{1}=\mathcal{M}_{1} \mathcal{M}$ and $\mathcal {C}_{1}=\mathcal{M}_{1} \mathcal{N}+\mathcal{N}_{1}$. 
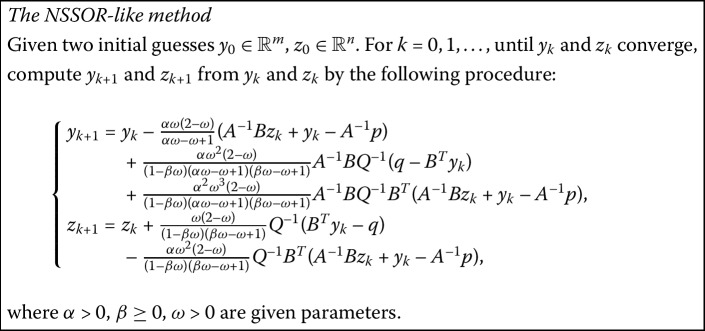


### The generalized NSOR-like method (GNSOR-like)

By introducing a diagonal matrix $\Omega=\operatorname{diag}(\omega I_{m}, \tau I_{n})$, where $I_{m}$, $I_{n}$, and further *I* are all identity matrices, we can obtain a generalization of the NSOR-like method:

$$\left ( \textstyle\begin{array}{c} y_{k+1} \\ z_{k+1} \end{array}\displaystyle \right )= (\mathcal{D}-\Omega \mathcal{L})^{-1}\bigl((I-\Omega)\mathcal{D}+\Omega \mathcal{U}\bigr) \left ( \textstyle\begin{array}{c} y_{k} \\ z_{k} \end{array}\displaystyle \right )+(\mathcal{D}-\Omega \mathcal{L})^{-1}\Omega \left ( \textstyle\begin{array}{c} p \\ -q \end{array}\displaystyle \right ). $$

More precisely, we have the following algorithmic description of the GNSOR-like method. 
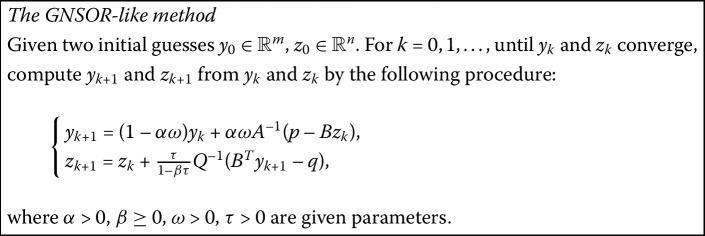


#### Remark

This method, in spirit, is analogous to the GSOR method [5]. It uses a relaxation matrix Ω for the NSOR-like method instead of a single relaxation parameter. Obviously, when $\omega=\tau$, this method reduces to the NSOR-like method mentioned in the previous section.

### The generalized NSSOR-like method (GNSSOR-like)

Ordinarily, the GNSSOR-like method can also be derived by combining the symmetric technique, as introduced in [25]. 
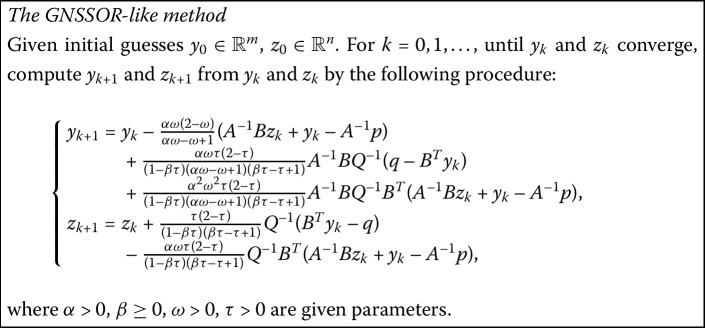


#### Remark

It can be seen easily that, when $\omega=\tau$, this method reduces to the NSSOR-like method mentioned in the previous subsection.

With different choices of parameters, the GNSSOR-like method covers several SSOR methods as follows:

(i) When $\alpha=1$ and $\beta=0$, we get the SSOR method in [10], 
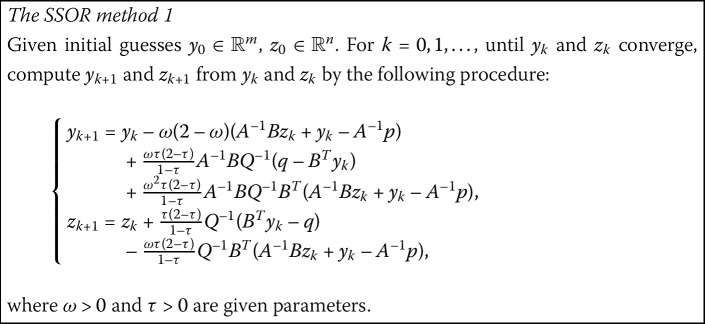
(ii) When $\alpha=1$ and $\beta=1/2$, we get the SSOR method in [23], 
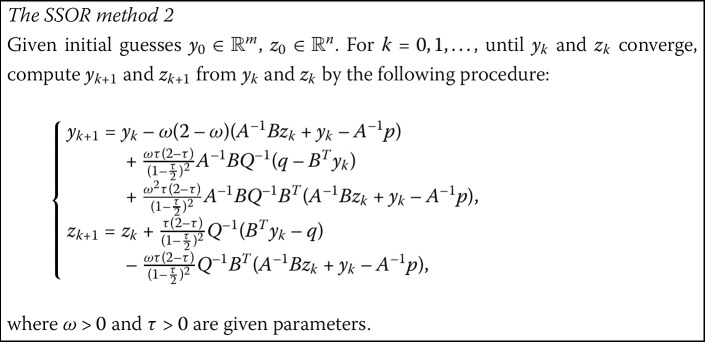

## Convergence analysis

In the section, we give a convergence analysis of these methods. We need the following lemmas.

### Lemma 3.1

(see [24])

*For the real quadratic equation* $x^{2}-bx+c=0$, *both roots are less than one in modulus if and only if* $\vert c \vert <1$ *and* $\vert b \vert <1+c$.

### Lemma 3.2

*Let* $\mathcal{R}$ *be the iteration matrix of the GNSOR*-*like method*, *and let* *λ* *be an eigenvalue of the matrix* $\mathcal{R}$. *Then* $\lambda \neq1$.

### Proof

Suppose on the contrary that $\lambda=1$ is an eigenvalue of the iteration matrix $\mathcal{R}$ and that the corresponding eigenvector is $(y^{T},z^{T})^{T}$. Then we have

$$\mathcal{R}\left ( \textstyle\begin{array}{c} y \\ z \end{array}\displaystyle \right ) =\left ( \textstyle\begin{array}{c} y \\ z \end{array}\displaystyle \right ), $$

or

$$\left ( \textstyle\begin{array}{c@{\quad}c} \frac{1}{\alpha}A & 0 \\ -\tau B^{T} & (1-\tau\beta) Q \end{array}\displaystyle \right ) \left ( \textstyle\begin{array}{c} y \\ z \end{array}\displaystyle \right ) =\left ( \textstyle\begin{array}{c@{\quad}c} (\frac{1}{\alpha}-\omega)A & -\omega B \\ 0 & (1-\tau\beta) Q \end{array}\displaystyle \right ) \left ( \textstyle\begin{array}{c} y \\ z \end{array}\displaystyle \right ). $$

From this equation we can deduce that

$$B^{T}y=0,\qquad Ay+Bz=0. $$

Thus $y=-A^{-1}Bz$ and $B^{T}A^{-1}Bz=0$. Since the matrix $B^{T}A^{-1}B$ is symmetric positive definite, we have $z=0$. Hence we get $y=0$, a contradiction! □

### Theorem 3.3

*Let the matrix* *A* *be symmetric positive definite for the saddle*-*point problem* (1.2), *and let the matrix* *B* *be of full column rank*. *Let* $\mathcal{R}$ *be the iteration matrix of the GNSOR*-*like method*. *Then the GNSOR*-*like method is convergent if the parameters* *α*, *β*, *ω*, *and* *τ* *satisfy*

$$0< \omega, \tau< 2,\qquad0< \alpha< \frac{2}{\omega}, \qquad0< \frac {\alpha\rho\omega\tau}{1-\beta\tau}< 2(2- \alpha\omega), $$

*where* *ρ* *denotes the spectral radius of the matrix* $Q^{-1}B^{T}A^{-1}B$.

### Proof

Evidently, we can see that the eigenvalues of the matrix $Q^{-1}B^{T}A^{-1}B$ are all real and positive. Let *λ* be a nonzero eigenvalue of the iteration matrix $\mathcal{R}$, and let $\bigl( {\scriptsize\begin{matrix}{}y \cr z \end{matrix}} \bigr)$ be the corresponding eigenvector. Then we have

$$\mathcal{R}\left ( \textstyle\begin{array}{c} y \\ z \end{array}\displaystyle \right ) =\lambda \left ( \textstyle\begin{array}{c} y \\ z \end{array}\displaystyle \right ) $$

or, equivalently,

$$\lambda \left ( \textstyle\begin{array}{c@{\quad}c} \frac{1}{\alpha}A & 0 \\ -\tau B^{T} & (1-\tau\beta) Q \end{array}\displaystyle \right ) \left ( \textstyle\begin{array}{c} y \\ z \end{array}\displaystyle \right ) =\left ( \textstyle\begin{array}{c@{\quad}c} (\frac{1}{\alpha}-\omega)A & -\omega B \\ 0 & (1-\tau\beta) Q \end{array}\displaystyle \right ) \left ( \textstyle\begin{array}{c} y \\ z \end{array}\displaystyle \right ). $$

Computations show that

3.1 $$ \left\{ \textstyle\begin{array}{l} (1-\lambda-\alpha\omega)Ay=\alpha \omega Bz, \\ (\lambda-1) (1-\tau\beta)Qz=\lambda\tau B^{T}y. \end{array}\displaystyle \right . $$

We get $(1-\lambda-\alpha\omega)y=\alpha\omega A^{-1}Bz$ from the first equality in (3.1), and hence $\lambda\tau(1-\lambda-\alpha\omega)B^{T}y=\lambda\tau \alpha\omega B^{T}A^{-1}Bz$ when $\lambda\neq1-\alpha\omega$. From this equality and the second equality in (3.1) it follows that

$$\lambda\tau\alpha\omega Q^{-1}B^{T}A^{-1}Bz=( \lambda-1) (1-\tau\beta ) (1-\lambda-\alpha\omega)z. $$

If $\lambda=1-\alpha\omega\neq0$, then $Bz=0$ and $-\alpha\omega (1-\tau\beta)Qz=\lambda\tau B^{T}y$. It then follows that $z=0$ and $y\in\operatorname{null}(B^{T})$, where $\operatorname{null}(B^{T})$ is the null space of the matrix $B^{T}$. Hence $\lambda=1-\alpha\omega$ is an eigenvalue of the matrix $\mathcal{R}$ with the corresponding eigenvector $(y^{T},0)^{T}$ with $y\in\operatorname{null}(B^{T})$.

Therefore, except for $\lambda=1-\alpha\omega$, the rest of the eigenvalues *λ* of the matrix $\mathcal{R}$ and all the eigenvalues *μ* of the matrix $Q^{-1}B^{T}A^{-1}B$ are of the functional relationship

$$\lambda\alpha\omega\tau\mu=(\lambda-1) (1-\tau\beta) (1-\lambda -\alpha\omega), $$

that is, *λ* satisfies the quadratic equation

3.2 $$ \lambda^{2}- \biggl(2-\alpha\omega-\frac{\alpha\mu\omega\tau }{1-\tau\beta} \biggr)\lambda+1-\alpha\omega=0. $$

By Lemma 3.1 we know that $\lambda=1-\alpha\omega$ and both roots *λ* of Eq. (3.2) satisfy $\vert \lambda \vert <1$ if and only if

$$\vert 1-\alpha\omega \vert < 1,\qquad \biggl\vert 2-\alpha\omega- \frac{\alpha\mu \omega\tau}{1-\beta\tau} \biggr\vert < 2-\alpha\omega. $$

Thus we can deduce that

$$0< \alpha< \frac{2}{\omega}, \qquad0< \frac{\alpha\rho\omega\tau }{1-\beta\tau}< 2(2-\alpha\omega), $$

where *ρ* denotes the spectral radius of the matrix $Q^{-1}B^{T}A^{-1}B$.

The proof of the theorem has been completed. □

### Lemma 3.4

*Let* $\mathcal{T}$ *be the iteration matrix of the GNSSOR*-*like method*. *Then we have*:

(i) $\lambda=\frac{(1-\omega+\omega\alpha)-\omega\alpha (2-\omega)}{1-\omega+\omega\alpha}$ *is an eigenvalue of the matrix* $\mathcal{T}$ *with multiplicity* $m-n$ *at least*.
(ii) *A real number* $\lambda\neq\frac{(1-\omega+\omega \alpha)-\omega\alpha(2-\omega)}{1-\omega+\omega\alpha}$ *is an eigenvalue of the matrix* $\mathcal{T}$ *if and only if there exists a real eigenvalue* *μ* *of the matrix* $Q^{-1}B^{T}A^{-1}B$ *such that* *λ* *is a zero point of*
  3.3 $$ \begin{aligned}[b] &(\lambda-1) (1-\tau+\beta\tau) (1-\beta\tau)\biggl[\biggl( \frac{1}{\alpha }-\omega\biggr) (1-\omega)-\lambda\biggl(\frac{1}{\alpha}+\omega- \frac{\omega}{\alpha }\biggr)\biggr]\\&\quad{}-\lambda\omega\tau(2-\omega) (2-\tau)\mu.\end{aligned} $$

### Proof

Let $\lambda\neq0$ be an eigenvalue of the matrix $\mathcal{T}$, and let $u=\bigl( {\scriptsize\begin{matrix}{} y \cr z \end{matrix}} \bigr)$ be the corresponding eigenvector. Then we have

$$\mathcal{T}u =\lambda u $$

or, equivalently,

$$(1-\lambda) (\mathcal{D}-\Omega\mathcal{U})u=(2I-\Omega)\mathcal {D}( \mathcal{D}-\Omega\mathcal{L})^{-1}\Omega Au. $$

Computations show that

3.4 $$ \bigl[(1-\lambda) (1-\omega+\omega\alpha)-\omega\alpha(2-\omega ) \bigr]Ay=\omega\alpha(\lambda+1-\omega)Bz, $$

and we obtain that

3.5 $$ (1-\lambda) (1-\tau+\beta\tau) (1-\beta\tau)z-\omega\alpha\tau (2- \tau)Q^{-1}B^{T}A^{-1}Bz= \tau(2-\tau) (\alpha \omega-1) Q^{-1}B^{T}y. $$

Let $\lambda=\frac{(1-\omega+\omega\alpha)-\omega\alpha(2-\omega )}{1-\omega+\omega\alpha}$. From (3.4) we have $A^{-1}Bz=0$.

We get $B^{T}y=0$ and $z=0$ since the matrix *A* is symmetric positive definite and the matrix *B* is of full column rank. Note that $\operatorname{rank}(B^{T}) = n$, and then there exist $m-n$ independent nonzero solutions of $B^{T}y=0$, that is, there exist $m-n$ corresponding eigenvectors of $\lambda=\frac{(1-\omega+\omega\alpha )-\omega\alpha(2-\omega)}{1-\omega+\omega\alpha} $.

When $\lambda\neq\frac{(1-\omega+\omega\alpha)-\omega\alpha (2-\omega)}{1-\omega+\omega\alpha}$, from (3.4) we have

$$y=\frac{\alpha\omega(\lambda+1-\omega)}{(1-\lambda)(1-\omega +\alpha\omega)-\alpha\omega(2-\omega)}A^{-1}Bz. $$

Substituting *y* into (3.5), we get

$$\begin{gathered} (1-\lambda) (1-\tau+\beta\tau) (1-\beta\tau)\frac{1}{\alpha}z \\\quad= \biggl[\omega \tau(2-\tau)-\frac{\omega\tau(2-\tau)(1-\alpha \omega)(1-\omega+\lambda)}{ (1-\lambda)(1-\omega+\alpha\omega)-\alpha\omega(2-\omega )} \biggr]Q^{-1}B^{T}A^{-1}Bz\end{gathered} $$

or, equivalently,

$$\begin{gathered} (1-\lambda) (1-\tau+\beta\tau) (1-\beta\tau)\frac{1}{\alpha }\biggl[(1-\lambda) \biggl((1-\omega)\frac{1}{\alpha}+\omega\biggr)-\omega(2-\omega)\biggr]z \\ \quad=\omega\tau(2-\tau)\biggl\{ \biggl[(1-\lambda) \biggl((1-\omega) \frac {1}{\alpha}+\omega\biggr)-\omega(2-\omega)\biggr]-\biggl(\omega-\frac{1}{\alpha}\biggr) (\omega-\lambda-1)\biggr\} \\ \qquad{}\times Q^{-1}B^{T}A^{-1}Bz .\end{gathered} $$

Since *μ* is an eigenvalue of the matrix $Q^{-1}B^{T}A^{-1}B$, we have

$$\begin{gathered} (1-\lambda) (1-\tau+\beta\tau) (1-\beta\tau)\frac{1}{\alpha }\biggl[(1-\lambda) \biggl((1-\omega)\frac{1}{\alpha}+\omega\biggr)-\omega(2-\omega)\biggr]\\\quad =\omega \tau(2-\tau)\biggl\{ \biggl[(1-\lambda) \biggl((1-\omega)\frac{1}{\alpha }+\omega \biggr)-\omega(2-\omega)\biggr]-\biggl(\omega-\frac{1}{\alpha}\biggr) (\omega - \lambda-1)\biggr\} \mu;\end{gathered} $$

simply,

$$\begin{gathered} (\lambda-1) (1-\tau+\beta\tau) (1-\beta\tau)\biggl[\biggl(\frac{1}{\alpha }- \omega\biggr) (1-\omega)-\lambda\biggl(\frac{1}{\alpha}+\omega - \frac{\omega}{\alpha}\biggr)\biggr]\\\quad=\lambda\omega\tau(2-\omega) (2-\tau)\mu.\end{gathered} $$

Conversely, we can also trivially prove the following: □

### Theorem 3.5

*Let the matrix* *A* *be symmetric positive definite*, *and let the matrix B* *be of full column rank in Eq*. (1.2). *Assume that* *α*, *β*, *and* *ω* *satisfy* $(1-\beta\tau)(1-\tau+\beta\tau)(1-\omega+\omega\alpha)\neq0$. *We choose a nonsingular matrix* *Q* *such that all eigenvalues of the matrix* $Q^{-1}B^{T}A^{-1}B$ *are real*. *Let* $\mu_{\max}$, $\mu_{\min}$ *be the largest and the smallest eigenvalues of the matrix* $Q^{-1}B^{T}A^{-1}B$, *respectively*. *Then*:

(i) *If* $\mu_{\min}>0$, *then the GNSSOR*-*like method is convergent if and only if*
  $$\begin{aligned}& 0< \omega< 2;\qquad 0< \tau< 2 ; \\& \left \{ \textstyle\begin{array}{l@{\quad}l} \frac{1}{\alpha}>\frac{\omega^{2}}{2(\omega-1)},& \textit{if } 0< \omega< 1,\\ \frac{1}{\alpha}< \frac{\omega^{2}}{2(\omega-1)}, &\textit{if } 1< \omega< 2; \end{array}\displaystyle \right . \\& (1-\tau+\beta\tau) (1-\beta\tau) > 0 ; \\& \frac{\alpha\omega\tau(2-\omega)(2-\tau)\mu_{\max}}{(1-\tau +\beta\tau)(1-\beta\tau)(1-\omega+\omega\alpha)} < 2 \biggl[1+\frac{(1-\alpha\omega)(1-\omega)}{1+\alpha\omega-\omega } \biggr]. \end{aligned}$$
(ii) *If* $\mu_{\max}<0$, *then the GNSSOR*-*like method is convergent if and only if*
  $$\begin{aligned}& 0< \omega< 2 ; \qquad 0< \tau< 2 ; \\& \left \{ \textstyle\begin{array}{l@{\quad}l} \frac{1}{\alpha}>\frac{\omega^{2}}{2(\omega-1)},& \textit{if } 0< \omega< 1,\\ \frac{1}{\alpha}< \frac{\omega^{2}}{2(\omega-1)}, &\textit{if } 1< \omega< 2; \end{array}\displaystyle \right . \\& (1-\tau+\beta\tau) (1-\beta\tau) < 0 ; \\& \frac{\alpha\omega\tau(2-\omega)(2-\tau)\mu_{\min}}{(1-\tau +\beta\tau)(1-\beta\tau)(1-\omega+\omega\alpha)} < 2 \biggl[1+\frac{(1-\alpha\omega)(1-\omega)}{1+\alpha\omega-\omega } \biggr]. \end{aligned}$$

### Proof

From (3.3) we get that

$$\lambda^{2}-\lambda \biggl[2-\frac{\omega\alpha(2-\omega )}{1+\alpha\omega-\omega}-\frac{\alpha\omega\tau(2-\omega )(2-\tau)\mu}{ (1-\tau+\beta\tau)(1-\beta\tau)(1-\omega+\omega\alpha)} \biggr] + \biggl(1-\frac{\omega\alpha(2-\omega)}{1+\alpha\omega-\omega } \biggr)=0. $$

By Lemma 3.1, $\vert \lambda \vert <1$ if and only if

3.6 $$ \biggl\vert \frac{(1-\alpha\omega)(1-\omega)}{1+\alpha\omega-\omega } \biggr\vert < 1 $$

and

$$\biggl\vert \frac{(1-\alpha\omega)(1-\omega)}{1+\alpha\omega-\omega }+1-\frac{\alpha\omega\tau(2-\omega)(2-\tau)\mu}{ (1-\tau+\beta\tau)(1-\beta\tau)(1+\alpha\omega-\omega)} \biggr\vert < 1+ \frac{(1-\alpha\omega)(1-\omega)}{1+\alpha\omega-\omega}. $$

Computations show that

$$\begin{gathered} \frac{\alpha\omega\tau(2-\omega)(2-\tau)\mu}{ (1-\tau+\beta\tau)(1-\beta\tau)(1+\alpha\omega-\omega)} > 0, \\ \frac{\alpha\omega\tau(2-\omega)(2-\tau)\mu}{ (1-\tau+\beta\tau)(1-\beta\tau)(1+\alpha\omega-\omega)} < 2+\frac{(1-\alpha\omega)(1-\omega)}{1+\alpha\omega-\omega};\end{gathered} $$

if $\mu_{\min}>0$, then we have

$$\begin{gathered} (1-\tau+\beta\tau) (1-\beta\tau) (1+\alpha\omega-\omega)>0, \\ \begin{aligned} 0 & < \frac{\alpha\omega\tau(2-\omega)(2-\tau)\mu}{ (1-\tau+\beta\tau)(1-\beta\tau)(1+\alpha\omega-\omega)} \\ & \leq \frac{\alpha\omega\tau(2-\omega)(2-\tau)\mu_{\max}}{ (1-\tau+\beta\tau)(1-\beta\tau)(1+\alpha\omega-\omega)} \\ & < 2+\frac{2(1-\alpha\omega)(1-\omega)}{1+\alpha\omega-\omega};\end{aligned}\end{gathered} $$

if $\mu_{\max}<0$, then we have

$$\begin{aligned}& (1-\tau+\beta\tau) (1-\beta\tau) (1+\alpha\omega-\omega)< 0, \\& \begin{aligned} 0 & < \frac{\alpha\omega\tau(2-\omega)(2-\tau)\mu}{ (1-\tau+\beta\tau)(1-\beta\tau)(1+\alpha\omega-\omega)} \\ & \leq \frac{\alpha\omega\tau(2-\omega)(2-\tau)\mu_{\min}}{ (1-\tau+\beta\tau)(1-\beta\tau)(1+\alpha\omega-\omega)} \\ & < 2+\frac{2(1-\alpha\omega)(1-\omega)}{1+\alpha\omega-\omega}.\end{aligned} \end{aligned}$$

From (3.6) we have

$$\begin{gathered} \frac{1}{\alpha}>\frac{\omega^{2}}{2(\omega-1)},\quad\hbox{if }0< \omega< 1, \\ \frac{1}{\alpha}< \frac{\omega^{2}}{2(\omega-1)},\quad\hbox{if } 1< \omega< 2.\end{gathered} $$

Cosidering $1+\alpha\omega-\omega>0$, we obtain

$$\left \{ \textstyle\begin{array}{l@{\quad}l} \frac{1}{\alpha}>\frac{\omega^{2}}{2(\omega-1)}, &\hbox{if } 0< \omega< 1,\\ \frac{1}{\alpha}< \frac{\omega^{2}}{2(\omega-1)},&\hbox{if } 1< \omega< 2. \end{array}\displaystyle \right . $$

 □

## Numerical experiments

In this section, we test several experiments to show the effectiveness of the GNSOR-like method and compare it with the SOR-like method in [13], MSOR-like method in [19], and MSOR-like method in [15]. We present computational results in terms of the numbers of iterations (denoted by IT) and computing time (denoted by CPU). We denote the choices of parameters *α*, *β*, *ω*, *τ* by $\alpha_{\exp}$, $\beta_{\exp}$, $\omega_{\exp}$, $\tau_{\exp}$ in our test, respectively. In our implementations, all iterations are run in MATLAB R2015a on a personal computer and are terminated when the current iterate satisfies $\mathit{RES}<10^{-6}$ or the number of iterations is more than 1000. In our experiments, the residue is defined to be

$$\mathit{RES}:=\sqrt{ \Vert Ay_{k}+Bz_{k}-p \Vert ^{2}+ \bigl\Vert B^{T}y_{k}-q \bigr\Vert ^{2}}< 10^{-6}, $$

the right-hand-side vector $(p^{T},q^{T})^{T}=\mathcal{A}e$ with $e=(1,1,\ldots,1)^{T}$, and the initial vectors are set to be $y_{0}=(0,0,\ldots,0)^{T}$ and $z_{0}=(0,0,\ldots,0)^{T}$.

### Example 4.1

([4])

Consider the saddle-point problem (1.1) in which

$$A=\left ( \textstyle\begin{array}{c@{\quad}c} I\otimes T+T\otimes I & 0\\ 0 & I\otimes T+T\otimes I \end{array}\displaystyle \right )\in\mathbb{R}^{2l^{2}\times2l^{2}}, \qquad B=\left ( \textstyle\begin{array}{c} I\otimes F\\ F\otimes I \end{array}\displaystyle \right )\in \mathbb{R}^{2l^{2}\times l^{2}}, $$

and

$$T=\frac{1}{h^{2}}\operatorname{tridiag}(-1,2,-1)\in\mathbb{R}^{l\times l},\qquad F= \frac{1}{h}\operatorname{tridiag}(-1,1,0)\in\mathbb{R}^{l\times l}, $$

where $h=\frac{1}{l+1}$ is the mesh size, and ⊗ be the Kronecker product.

In the test, we set $m=2l^{2}$ and $n=l^{2}$. The choices of the matrix *Q* are listed in Table 1 for Example 4.1. We have the following computational results summarized in Table 2.

**Table 1:** Choices of matrix *Q* for Example 4.1

	Matrix *Q*	Description
Case I	$B^{T}\hat{A}^{-1}B$	*Â* = diag(*A*)
Case II	$B^{T}\hat{A}^{-1}B$	*Â* = tridiag(*A*)

**Table 2:** ITs and CPUs of SOR-like, MSOR-like [15], NSOR-like [14], GNSOR-like, and NSSOR-like for Example 4.1

*m*			128	512	1152	2048	8192
*n*			64	256	576	1024	4096
*m* + *n*			192	768	1728	3072	12,288
Case I	SOR-like	$\omega_{\exp }$	0.4644	0.2720	0.1886	0.1386	0.0741
		IT	73	202	221	315	657
		CPU	0.0284	0.1321	0.3670	0.9262	9.0859
	MSOR-like [15]	$\alpha_{\exp}$	1.7212	1.8256	1.8737	1.9136	1.9201
		$\omega_{\exp}$	0.3159	0.1873	0.1328	0.1020	0.0541
		IT	67	140	183	223	462
		CPU	0.0313	0.1171	0.2937	0.6137	6.3687
	NSOR-like [14]	$\alpha_{\exp}$	1.7212	1.8256	1.8689	1.9699	1.9219
		$\beta_{\exp}$	0.3700	0.3655	0.3492	0.2399	0.2148
		$\omega_{\exp}$	0.3159	0.1873	0.1328	0.1001	0.0545
		IT	55	105	160	212	457
		CPU	0.0250	0.1031	0.2624	0.5687	6.2634
	GNSOR-like	$\alpha_{\exp}$	1.7212	1.8256	1.8689	1.9699	1.9219
		$\beta_{\exp}$	0.3700	0.3655	0.3492	0.2399	0.2148
		$\omega_{\exp}$	0.3250	0.1923	0.1361	0.1029	0.0555
		$\tau_{\exp}$	0.3130	0.1830	0.1282	0.0985	0.0534
		IT	50	101	155	211	447
		CPU	0.0154	0.1019	0.2561	0.5663	6.1488
Case II	SOR-like	$\omega_{\exp }$	0.5958	0.3657	0.2215	0.1961	0.0945
		IT	56	103	183	216	509
		CPU	0.0310	0.1343	0.5855	1.4298	16.1417
	MSOR-like [15]	$\alpha_{\exp}$	1.6599	1.7732	1.8315	1.8753	1.9200
		$\omega_{\exp}$	0.3996	0.2498	0.1806	0.1257	0.0745
		IT	45	94	167	175	330
		CPU	0.0306	0.1194	0.6196	1.2068	10.4589
	NSOR-like [14]	$\alpha_{\exp}$	1.6469	1.7582	1.8318	1.8750	1.9100
		$\beta_{\exp}$	0.3397	0.3438	0.3640	0.3541	0.4001
		$\omega_{\exp}$	0.3986	0.2513	0.1812	0.1420	0.0758
		IT	39	77	116	155	320
		CPU	0.0164	0.0916	0.4162	1.0173	9.4823
	GNSOR-like	$\alpha_{\exp}$	1.6469	1.7582	1.8318	1.8750	1.9100
		$\beta_{\exp}$	0.3397	0.3438	0.3640	0.3541	0.4001
		$\omega_{\exp}$	0.4030	0.2513	0.1825	0.1402	0.0758
		$\tau_{\exp}$	0.4210	0.2581	0.1823	0.1443	0.0755
		IT	37	75	115	154	319
		CPU	0.0134	0.0871	0.3219	1.0129	9.4803

From Table 2 we can see that the GNSOR-like method requires much less iteration number than NSOR-like [14], SOR-like, MSOR-like [15], so that it costs less CPU time than the others. So, the GNSOR-like method is effective for solving the saddle-point problem (1.1).

## Concluding remark

In this paper, we first presented several iteration methods for solving the saddle-point problem (1.1) and then give their convergence results. The GNSOR-like method is faster and requires much less iteration steps than the other methods mentioned in the paper.
